# Anal cancer and human papillomaviruses in heterosexual men

**DOI:** 10.3747/co.v15i5.295

**Published:** 2008-10

**Authors:** A. Nyitray

**Affiliations:** Richard J. Ablin, PhD, Research Professor of Immuno-biology and Pathology, University of Arizona College of Medicine and the Arizona Cancer Center, Tucson, Arizona, U.S.A. and Phil Gold, PhD MD, Professor of Medicine, Physiology, and Oncology, McGill University, Montreal, Quebec, Canada, Section Editors

## BACKGROUND

Human papillomavirus (hpv) causes a variety of anogenital cancers and is considered the primary cause of anal canal cancer 1,2. Anal cancer is a rare disease: a total of 4650 diagnoses of anal carcinoma and 690 deaths were expected in the United States in 2007, with approximately 40% of the diagnoses and deaths being expected to occur in men. Other than cervical cancer, anal cancer was expected to be the most commonly diagnosed anogenital hpv-associated cancer in 2007 3.

## PARALLELS BETWEEN CERVICAL AND ANAL CANCER

Although anal cancer is less common than cervical cancer, its incidence in U.S. men is increasing, to 1.6 per 100,000 in 2005 from 0.6 per 100,000 in 1973. During the same period, the incidence of cervical cancer in women decreased 4. An increasing incidence of anal cancer noted also in Europe 5 is likely attributable to increased anal disease occurring in immunocompromised people and in men who have sex with men (msm) 6–8. Indeed, the risk ratio of observed to expected anal cancers has been reported to be 37.9 [95% confidence interval (ci): 33.0 to 43.4] in men with hiv, 59.5 (95% ci: 51.5 to 68.4) in homosexual men with hiv, and 5.9 (95% ci: 2.7 to 11.2) in heterosexual male injection drug users with hiv 9. Appropriately, anal cancer research to date has focused on these populations. However, it is a concern that healthy heterosexual men constitute 90% of the male population and that virtually no research regarding anal hpv has targeted them. Epidemiologic research also indicates that heterosexual men with hiv, even in the absence of receptive anal intercourse, may have a high prevalence of precancerous anal lesions associated with hpv^10^. Finally, a clearer understanding of the risks associated with anal hpv in heterosexual men may provide important contrasts that will help clarify the picture of anal hpv-related morbidity and mortality in other populations.

As in cervical cancer, most anal cancers are associated with oncogenic hpv types 16 and 18 2,11. Given an identical pathogen and similarity of the tissue (including a transformation zone in the anal canal that is bounded on each side by squamous and columnar epithelium 12), hpv-induced carcinogenesis leading to cervical cancer is comparable to the process that leads to anal canal cancer. However, although the deployment of a preventive vaccine for hpv infection and cervical dysplasia reflects important progress in preventing cases of cervical cancer 13, policymakers do not have the information needed to make informed decisions about vaccine delivery to prevent anal cancer. The necessary information will hopefully come from ongoing hpv vaccine studies in men and from modelling efforts that can provide quantitative insight into the economic and public health effects of the vaccine. However, accurate modeling of vaccine cost-effectiveness with regard to anal hpv in men requires more natural history data 14,15, and there are no such studies, published or ongoing, of anal hpv in heterosexual men. Indeed, few studies have estimated anal hpv prevalence in heterosexual men.

## PREVALENCE OF ANAL HPV

In a community-based study of anal hpv in 222 asymptomatic heterosexual men, we recently estimated anal hpv prevalence at 16.6% for the anal canal and 21.3% for the perianal region. Of men with anal hpv, one third harboured an oncogenic hpv type. Also, men with anal hpv were more than twice as likely as men with no anal hpv to be in their 20s [odds ratio (or): 2.41; 95% ci: 1.05 to 5.55] 16. Two other smaller studies of exclusively heterosexual men from a clinic for sexually transmitted infections and of partners of women with confirmed hpv estimated anal hpv prevalences of 1.2% 17 and 8% 18 respectively. The higher prevalence found in our sample may result from the use of more sensitive test kits for hpv dna detection.

These prevalence data are a first step in understanding anal hpv in heterosexual men, but they need to be confirmed in other populations of heterosexual men. We are currently conducting a three-country anal hpv prevalence study with men recruited by a collaborative team led by Dr. Anna Giuliano of the H. Lee Moffitt Cancer Center and Research Institute. Follow-up longitudinal studies are needed, because these prevalence data can blend (and obscure) critical natural history characteristics of anal hpv that are needed to inform prevention strategies.

## SUMMARY

Little is known about anal hpv in men—and especially in heterosexual men. Against this backdrop of scarce data, the incidence of anal cancer has risen considerably. Although knowledge about anal hpv in msm has advanced in the last decade (important advances, given the increased rate of anal cancer in msm), the much greater number of heterosexual men and the lack of epidemiologic data describing anal hpv in heterosexual men should prompt additional research in this area.
